# Development of an RF-EMF Exposure Surrogate for Epidemiologic Research

**DOI:** 10.3390/ijerph120505634

**Published:** 2015-05-22

**Authors:** Katharina Roser, Anna Schoeni, Alfred Bürgi, Martin Röösli

**Affiliations:** 1 Swiss Tropical and Public Health Institute, Socinstrasse 57, P.O. Box, CH-4002 Basel, Switzerland; E-Mails: katharina.roser@unibas.ch (K.R.); anna.schoeni@unibas.ch (A.S.); 2 University of Basel, Petersplatz 1, CH-4003 Basel, Switzerland; 3 ARIAS umwelt.forschung.beratung, Gutenbergstrasse 40B, CH-3011 Bern, Switzerland; E-Mail: alfred.buergi@arias.ch

**Keywords:** exposure assessment, RF-EMF, mobile phone, adolescents, dose calculation

## Abstract

Exposure assessment is a crucial part in studying potential effects of RF-EMF. Using data from the HERMES study on adolescents, we developed an integrative exposure surrogate combining near-field and far-field RF-EMF exposure in a single brain and whole-body exposure measure. Contributions from far-field sources were modelled by propagation modelling and multivariable regression modelling using personal measurements. Contributions from near-field sources were assessed from both, questionnaires and mobile phone operator records. Mean cumulative brain and whole-body doses were 1559.7 mJ/kg and 339.9 mJ/kg per day, respectively. 98.4% of the brain dose originated from near-field sources, mainly from GSM mobile phone calls (93.1%) and from DECT phone calls (4.8%). Main contributors to the whole-body dose were GSM mobile phone calls (69.0%), use of computer, laptop and tablet connected to WLAN (12.2%) and data traffic on the mobile phone via WLAN (6.5%). The exposure from mobile phone base stations contributed 1.8% to the whole-body dose, while uplink exposure from other people’s mobile phones contributed 3.6%. In conclusion, the proposed approach is considered useful to combine near-field and far-field exposure to an integrative exposure surrogate for exposure assessment in epidemiologic studies. However, substantial uncertainties remain about exposure contributions from various near-field and far-field sources.

## 1. Introduction

Mobile phones and other wireless communication devices using radiofrequency electromagnetic fields (RF-EMF) are an integral part in the everyday life of adolescents. Thus exposure to this radiation is ubiquitous and in studying potential effects of RF-EMF, exposure assessment is a crucial part in this field of research. Since there are a lot of different sources emitting RF-EMF in everyday life, one needs to find a way of combining all of these emissions to one single integrative exposure measure. On one hand there are near-field sources such as mobile phones, computers, laptops and tablets emitting close to the body. On the other hand far-field sources such as fixed site transmitters (mobile phone base stations and broadcast transmitters), Wireless Local Area Network (WLAN) base stations, Digital Enhanced Cordless Telecommunications (DECT) base stations and other mobile phones in the surrounding area contribute to the environmental exposure. So far, little attempts have been made to combine these different types of exposure to one single integrative measure.

In a German study, personal measurements in adolescents and adults have been conducted during 24 h to estimate RF-EMF exposure [1,2,3]. This approach considered all exposure sources in the environment. However, it is time-consuming and personal measurements may not adequately record exposure from near-field sources because measured values depend highly on the distance between the emitting source and the measurement device, which is not necessarily the same as the distance between the emitting source and the body [4,5]. Other studies focussed on far-field exposures only by using propagation models for fixed site transmitters [6,7,8,9,10,11,12,13]. Frei *et al.* combined modelled RF-EMF exposure from fixed site transmitters at home with personal exposure relevant characteristics and behaviour to estimate personal RF-EMF exposure [14]. In this study, the presence of concrete walls and metal window frames were found to modify RF-EMF exposure from fixed site transmitters. Additional exposure relevant factors included ownership of a mobile phone, ownership of a WLAN at home and having a DECT base station in the bedroom or at the place where the person spent most of their time during the day, time spent at an external workplace and hours per week spent in a train, tram or bus. However, this exposure proxy focussed on far-field sources only and near-field sources were separately considered in their epidemiological analyses on non-specific symptoms of ill health and RF-EMF exposure [15,16]. In the framework of the Interphone study, estimations of RF energy absorbed in the brain from mobile phones were assessed [17]. Lauer *et al.* calculated organ-specific and whole-body RF-EMF proxies taking into account far-field exposure from different sources and near-field exposure from calls on the mobile phone and on the DECT phone using data collected between 2007 and 2009 in Switzerland [18]. However, these data may already be outdated because in the meantime mobile phones have been developed in the direction of multifunctional devices used not only for making calls and sending text messages, but for many additional activities such as browsing the internet, watching videos and gaming. Thus, the exposure predictors are expected to have changed considerably, and a comprehensive overview of relevant factors influencing the RF-EMF exposure emitted by near-field and far-field sources is still missing. The aim of this study was to determine these relevant factors and to develop an integrative exposure assessment method combining near-field and far-field sources for the brain as well as for the whole-body RF-EMF exposure for epidemiologic research. As a result we present cumulative RF-EMF dose for adolescents of a Swiss epidemiologic study called Health Effects Related to Mobile phonE use in adolescentS (HERMES).

## 2. Methods

### 2.1. Hermes Study

The HERMES study, a cohort study conducted in Central Switzerland, aimed to prospectively investigate whether the exposure to RF-EMF emitted by mobile phones and other wireless communication devices affects cognitive functions or causes behavioural problems and non-specific health disturbances in adolescents. The investigation took place from June 2012 to March 2013. The study participants filled in a paper-and-pencil questionnaire during school time supervised by two study managers. The questionnaire included detailed questions about their mobile phone use, DECT phone use and computer, laptop and tablet use (in brackets are the corresponding near-field exposure predictors indicated):

- Duration of calls made and received with their own and other mobile phones (GSM and UMTS mobile phone calls);
- Proportion of calls with the mobile phone using a headset (GSM and UMTS mobile phone calls);
- Duration of mobile phone use for data traffic (mobile phone data traffic and mobile phone data traffic WLAN);
- Duration of carrying the mobile phone close to the body (mobile phone close to body);
- Duration of calls made and received with a DECT phone at home (DECT phone calls);
- Duration of computer, laptop and tablet use and WLAN connection of the corresponding devices (computer, laptop and tablet use with WLAN).

Additionally, they were asked about the time spent in trains and buses. Furthermore we distributed paper-and-pencil questionnaires for the parents and asked them to return these directly to the study managers. This questionnaire included questions about DECT phones, WLAN and number of smartphones at home and number of floors and floor location of the residence. In addition, the teacher or head of the school provided us with information about the availability of WLAN in the school and building characteristics of the school building (number of floors and the floor location of the class room the adolescents spent most of their school time). Informed consent was given by the study participants and their parents to obtain objective mobile phone use data from the mobile phone operators. Operator data included records for each call made and received including duration of call and network used when starting the call. The calls were categorised into calls on the Global System for Mobile Communications (GSM) network and on the Universal Mobile Telecommunications System (UMTS) network. There was no differentiation between GSM900 and GSM1800 network in the mobile phone operator data. Average proportions of network use for calls over the recorded time period were used in our analysis.

### 2.2. Personal Measurements in the Framework of the Hermes Study

A subgroup of the study participants also took part in personal measurements. The adolescents carried a portable measurement device, a so-called exposimeter, for three consecutive days. Two versions of the device Expom (referred to as *Expom 1* for the older version and *Expom 3* for the newer version) were used to measure 13 frequency bands ranging from Digital Video Broadcasting—Terrestrial (DVB-T, centre frequency of 620 MHz) to Worldwide Interoperability for Microwave Access (WiMa, 3500 MHz) [19]. Nine out of the 13 measured frequency bands were used in our analysis (Table 1).

**Table 1:** Frequency range, quantitation limits and reporting limits for the frequency bands of the measurement devices Expom 1 and Expom 3 used for the personal measurements.

Frequency Band	Frequency Range (MHz)	Quantitation Limit (V/m)		Reporting Limit (V/m)
	Expom 1 and Expom 3	Expom 1	Expom 3	Expom 1 and Expom 3
TV	470–790	0.010	0.005	0.0025
Uplink 900 *	880–915	0.015	0.005	0.0025
Downlink 900 *	925–960	0.015	0.005	0.0025
Uplink 1800 *	1710–1785	0.015	0.005	0.0025
Downlink 1800 *	1805–1880	0.005	0.005	0.0025
DECT	1880–1900	0.005	0.005	0.0025
Uplink 1900 *	1920–1980	0.003	0.003	0.0015
Downlink 2100 *	2110–2170	0.010	0.003	0.0015
WLAN	2400–2485	0.005	0.005	0.0025

* The uplink and downlink bands include all technologies using the particular frequency range. Downlink means the transmission from mobile phone base stations to mobile phone handsets and uplink the transmission from mobile phone handsets to mobile phone base stations.

Additionally, the participants filled in a time-activity diary installed as an application on a smartphone operating in flight mode. These diaries were manually corrected for implausible chronologies of diary entries. Subsequently, summary statistics were calculated after censoring the measurements at the reporting limit and 5 V/m.

### 2.3. Dose Calculations

We aimed to calculate personal cumulative RF-EMF doses in the brain and the whole-body combining exposure from different sources emitting RF-EMF. The processes of learning and memory are located in the hippocampus, while processes for behaviour and cognitive functions in the prefrontal cortex. The hippocampus and the prefrontal cortex consist mainly of gray matter. Therefore, specific absorption rates (SARs) for *brain gray matter* were used for the brain exposure. Additionally, the same calculations were performed for *brain white matter* and compared with the brain exposure obtained for brain gray matter since the white matter is important for these processes as well.

The personal dose in terms of the time-averaged specific absorption rate (SAR) can be calculated as follows:

(1) $$\text{dose}=\underset{\text{i}}{\sum }{\text{dose}}_{\text{i}}=\underset{\text{i}}{\sum }{\text{SAR}}_{\text{i}}{\text{*time}}_{\text{i}}$$

with dose_i_ (in mJ/kg) and SAR_i_ (in mW/kg) the dose and SAR originating from the exposure in a certain frequency band or due to a certain use of a specific wireless communcation device, and time_i_ the duration of this exposure. Thus, the proposed integrative exposure surrogate consists of a near-field component combining the exposure from the use of wireless communication devices and a far-field component aggregating the exposure from environmental sources. Therefore, we calculated the total dose as follows:

(2) dose=near----field dose+far-field dose

#### 2.3.1. Near-Field Dose

For the near-field component, we considered *a priori* the following exposure predictors relevant:

(3) $$\text{near-field dose}={\text{dose}}_{\text{GSM mobile phone calls}}+{\text{dose}}_{\text{UMTS mobile phone calls}}+{\text{dose}}_{\text{DECT phone calls}}+{\text{dose}}_{\text{mobile phone data traffic}}+{\text{dose}}_{\text{mobile phone close to body}}+{\text{dose}}_{\text{mobile phone data traffic WLAN}}+{\text{dose}}_{\text{computer},\text{laptop and tablet use with WLAN}}$$

The particular dose parts of the near-field component of the exposure surrogate (Equation (3)) can be calculated as follows:

(4) $${\text{near-field dose}}_{\text{i}}={\text{SAR}}_{\text{i}}\left(\text{literature}\right)\;\times {\text{ time}}_{\text{i}}\left(\text{HERMES questionnaire},\text{ operator data}\right)$$

where the SAR_i_ were derived from the literature [18,20,21,22,23,24,25] and the exposure durations time_i_ were asked in the HERMES questionnaire. For participants with missing operator data, the proportion of network used for calls (GSM and UMTS) was estimated by regression modelling using the available mobile phone operator data from a subgroup of the study participants.

##### Derivation of the SARs

For the derivation of the SARs for the exposure circumstances in Equation (3) we combined the SARs provided from Lauer *et al.* for calls on the mobile phone and on the DECT phone [18] with the measured uplink output power from Persson *et al.* [24], Gati *et al.* [21] and Huang *et al.* [23]. Additionally, we took into account the SAR ranges presented in the SEAWIND Final Summary Report (referred to as *SEAWIND report*) [20].

For calls with a mobile phone Lauer *et al.* provide a brain (the *brain gray matter* values were used, referred to as *brain*) SAR of 3.198 mW/kg and a whole-body SAR of 0.411 mW/kg for GSM900/GSM1800 calls based on output powers derived from Vrijheid *et al.* for GSM900 and GSM1800 [25] (Table 2). For UMTS calls Lauer *et al.* calculated a brain SAR of 0.023 mW/kg and a whole-body SAR of 0.003 mW/kg using output power values from Gati *et al.* [21] and assuming half of the calls in buildings and the other half outdoors.

On average the SAR decreased by a factor of 1000 by having the device approximately 20 cm away from the body compared to a device touching the body [20]. Therefore, we used a brain SAR of 3.198×10^−3^ mW/kg for GSM900/GSM1800 calls with headset and a brain SAR of 0.023 × 10^−3^ mW/kg for UMTS calls with headset. For the whole-body exposure we used the same SAR for calls with and without headset referring to a similar distance to the body when having the mobile phone close to the ear or in front of the body while using a headset. 

**Table 2:** Near-field *brain* and *whole-body* SARs, corresponding derivation and references for the near-field predictors.

Near-Field Predictor	Brain SAR		Whole-Body SAR		References
	(mW/kg)	Derivation	(mW/kg)	Derivation	
GSM ^1^ mobile phone calls without headset	3.198	−	0.411	−	[18]
GSM ^1^ mobile phone calls with headset	3.198 × 10^−3^	3.198 × 0.001	0.411	0.411 × 1	[18,20]
UMTS mobile phone calls without headset	0.023	−	0.003	−	[18]
UMTS mobile phone calls with headset	0.023 × 10^−3^	0.023 × 0.001	0.003	0.003 × 1	[18,20]
DECT phone calls without eco mode	0.373	−	0.051	−	[18]
DECT phone calls with eco mode	0.0373	0.373 × 0.1	0.0051	0.051 × 0.1	[18,20]
mobile phone data traffic with mobile internet connection	0.092 × 10^−3^	0.023 × 4 × 0.001	0.012	0.003 × 4 × 1	[18,20,21,22,23,24]
mobile phone close to body (passive mobile phone data traffic)	0.092 × 10^−3^	0.023 × 4 × 0.001	0.012	0.003 × 4 × 1	[18,20,21,22,23,24]
mobile phone data traffic with WLAN	0.092 × 10^−3^	0.023 × 4 × 0.001	0.012	0.003 × 4 × 1	[18,20,21,22,23,24]
computer, laptop and tablet use with WLAN	0.092 × 10^−3^	0.023 × 4 × 0.001	0.012	0.003 × 4 × 1	[18,20,21,22,23,24]

**^1^** For calls with the mobile phone on the GSM network the mean of the SARs for the GSM900 and the GSM1800 network was used because there was no differentiation between GSM900 and GSM1800 network in the mobile phone operator data.

For DECT phone calls Lauer *et al.* derived an average output power from the general transmission power of a DECT phone [18], resulting in a brain SAR of 0.373 mW/kg and a whole-body SAR of 0.051 mW/kg. The SEAWIND report showed a decrease in the SAR by a factor of 10 for calls with an eco mode DECT phone compared to a DECT phone without eco mode [20], therefore we used a brain SAR of 0.0373 mW/kg and a whole-body SAR of 0.0051 mW/kg for calls with a DECT phone provided with eco mode.

For the output power of mobile phones during data transmission we took the following available knowledge into account: Persson *et al.* measured on average an increased output power for data connections compared to voice connections in the UMTS network in the range of a factor of 3.25 to 6.8, depending on rural or urban environment and the bit rates used for the data transmission [24]. In the framework of the LEXNET project an output power increased by about a factor of 4 for data traffic service compared to voice service in the 3G network of Orange in France was found [23]. Gati *et al.* found a mean output power increased by about 6 dB on average for data traffic mode compared to voice mode [21]. Therefore, we used a by a factor of 4 increased output power of the mobile phone for data traffic compared to calls on the UMTS network. To take into account the different positions of the mobile phone during data traffic compared to those during calls (holding the mobile phone in the hand instead of close to the ear) we used the ranges delivered in the SEAWIND report for different distances between the device and the respective tissue [20]. The SAR decreased on average by a factor of 1000 by having the device approximately 20 cm away from the body compared to a device touching the body. Hadjem *et al.* found that the exposure for a mobile phone in watching-like position at 10 cm distance is about ten times below the exposure in voice position. At 40 cm distance it appeared that the exposure was about 1000 times lower [22]. These findings are comparable with the SAR ranges presented in the SEAWIND report for UMTS voice and UMTS data [20]. These considerations led us to use a brain SAR of 0.092 × 10^−3^ mW/kg for data traffic on the mobile phone via mobile internet connection. For the whole-body SAR we assumed that the mobile phone is held approximately at the same distance from the body for data traffic as for voice calls resulting in a whole-body SAR of 0.012 mW/kg for data traffic on the mobile phone via mobile internet connection.

Considering an approximately equal exposure for transmission of a fixed size data packet using UMTS or WLAN (SEAWIND report, page 3 [20]) we decided to use the same SAR for data traffic via WLAN as for data traffic via mobile internet connection for both the brain and the whole-body SAR. 

For using a computer, laptop or tablet connected to the internet via WLAN we used the same SAR as for data traffic on the mobile phone using WLAN assuming approximately the same distance between the device and the brain and the body, respectively.

#### 2.3.2. Far-Field Dose

The far-field component consisted of the following parts:

(5) $$\begin{array}{ll} \text{far-field dose} & ={\text{dose}}_{\text{radio}}+{\text{dose}}_{\text{TV}}+{\text{dose}}_{\text{downlink }900}+{\text{dose}}_{\text{downlink }1800}\\ & +{\text{dose}}_{\text{downlink }2100}+{\text{dose}}_{\text{WLAN}}+{\text{dose}}_{\text{DECT}}+{\text{dose}}_{\text{uplink}} \end{array}$$

where downlink means the transmission from mobile phone base stations to mobile phone handsets and uplink the transmission from mobile phone handsets to mobile phone base stations.

Far-field exposure from radio and TV broadcast transmitters and mobile phone base stations at home and in school were considered a priori relevant and were modelled using a geospatial propagation model [9,10]. Additionally, behaviours and characteristics relevant for the remaining far-field exposure parts (WLAN, DECT and uplink; Equation (5)) were estimated from the personal measurements. Far-field dose parts were obtained by multiplying the estimated power flux density with the normalized organ and frequency specific SAR derived from the literature [18] and the exposure duration obtained from the HERMES questionnaire or from the diary filled in during the personal measurements:

(6) $$\begin{array}{ll} {\text{far-field dose}}_{\text{i}} & \\ & ={\text{SAR}}_{\text{i}}\left(\text{literature},\text{ modelling},\text{personal measurements}\right)\\ & \times {\text{ time}}_{\text{i}}\left(\text{HERMES questionnaire},\text{ personal measurements}\right) \end{array}$$

##### Geospatial Propagation Model

Far-field exposure from fixed site transmitters was modelled using a geospatial propagation model based on a comprehensive database of transmitters, three-dimensional topography and a three-dimensional building model of the study area [9,10]. The coordinates of the home and the school addresses of the study participants were provided from the Swiss Federal Statistical Office. The number of floors of the building and the floor location of the residence and class room for calculating the height of the residence and class room were asked in the parents’ and school questionnaire, respectively [9,14]. On average, a damping factor of 4.6 dB was used for outdoor-to-indoor modelling to take into account wall attenuation [9].

#### 2.3.3. Multivariable Regression Models

Behaviours resulting in far-field exposure from WLAN base stations, DECT base stations and uplink of other mobile phones in the surroundings were identified by means of multivariable regression models using non-parametric bootstrap to estimate the coefficients. In these models, personal measurements were used as dependent variables. The explanatory variables, such as time spent in rooms or buildings with WLAN or DECT base station, number of smartphones in the household, or time spent in public transport were derived from the HERMES questionnaire. Regression models were also used to evaluate whether building characteristics modified indoor personal exposure from fixed site transmitters as was previously observed [14].

##### Combining Near-Field and Far-Field Dose

Using the equations above, we calculated daily brain and whole-body RF-EMF dose for each HERMES study participant. For data visualisation we have additionally chosen three HERMES study participants: a non-user, a normal user and a heavy-user. The non-user is a study participant not owning a mobile phone and not using another mobile phone (12 out of 439 study participants reported not to use a mobile phone at all). The normal user is an average mobile phone call (median = 6.4 min/day) and data traffic user (median data traffic via mobile internet connection = 2.27 min/day, median data traffic via WLAN = 19.0 min/day). The heavy-user represents maximal duration of mobile phone calls (267.1 min/day) and average mobile phone data traffic use. Note that all three users are average HERMES users in terms of calls on the DECT phone at home and computer, laptop and tablet use with WLAN (duration of DECT phone calls and us of devices with WLAN close to the median of the study population, median duration of DECT phone calls = 4.8 min/day, median use of devices with WLAN = 30 min/day).

### 2.4. Comparison of Dose Calculations with Personal Measurements

For the 95 participants with personal measurements we compared the dose with the personal measurements. For brain and whole-body dose three exposure categories were defined: brain or whole-body dose <50th percentile (*low*), 50th–90th percentile (*medium*) and >90th percentile (*high*).

All subjects gave their informed consent for inclusion before they participated in the study. The study was conducted in accordance with the Declaration of Helsinki, and the protocol was approved by the Ethics Committee of Lucerne, Switzerland on 9 May, 2012 (Project identification code: EK 12025).

## 3. Results

Four hundred and thirty nine (439) adolescents with a mean age of 14.0 years (range: 12.1–17.0 years) took part in the HERMES study. Objectively recorded operator data was available for a subgroup of 233 study participants. After data cleaning of the personal measurements and diary cleaning, 95 out of 121 collected sets of measurements and diaries could be used in our analysis.

### 3.1. Near-Field Dose

#### 3.1.1. Near-Field Predictors

The adolescents of the HERMES study indicated in the questionnaire average mobile phone call duration of 17.2 min/day, of which 9.5 min were calls on the GSM network and 7.7 min calls on the UMTS network according to the recorded/predicted proportion of network use derived from the operator data (Table 3). They reported to use the DECT phone at home on for calls lasting 9.0 min per day. They used their mobile phone on average 11.5 min/day for data traffic on the mobile phone using a mobile internet connection and 30.6 min/day for data traffic using a WLAN connection. Additionally, they indicated to wear their mobile phone for 4.4 h close to the body. Lastly, they reported to use computers, laptops and tablets connected to the internet via WLAN for almost an hour per day (57.6 min).

#### 3.1.2. Near-Field Dose

**Figure 1:**
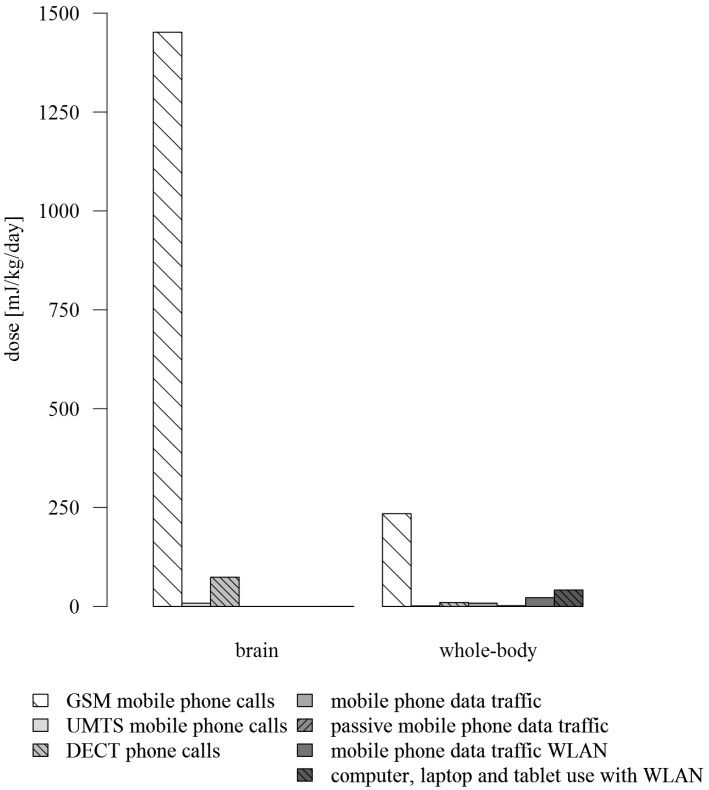
Mean daily cumulative *brain* (left) and *whole-body* (right) dose for the near-field predictors.

The highest dose rate (dose per 1 min) was found for calls on the mobile phone on the GSM network (without headset) with 191.88 mJ/kg/min and 24.66 mJ/kg/min followed by calls on the DECT phone (without eco mode) with 22.38 mJ/kg/min and 3.06 mJ/kg/min for brain and whole-body, respectively (Table 3). Considering all predictors, the brain near-field dose consisted mainly of the exposure from GSM mobile phone calls, on average 1451.78 mJ/kg/day (94.6%), followed by a dose of 74.10 mJ/kg/day (4.8%) from DECT phone calls (Table 3 and Figure 1). UMTS mobile phone calls counted for 8.04 mJ/kg/day (0.5%). Concerning the whole-body near-field dose, the largest part was induced by GSM mobile phone calls with a dose of 234.47 mJ/kg/day (73.3%). DECT phone calls contributed with a dose of 10.13 mJ/kg/day (3.2%). The dose contribution from mobile phone data traffic was 8.29 mJ/kg/day (2.6%) for data traffic via mobile internet connection and 22.03 mJ/kg/day (6.9%) for data traffic via WLAN connection. Using a computer, laptop and tablet connected to WLAN played a considerable role with a mean dose of 41.46 mJ/kg/day (13.0%).

**Table 3:** SAR, mean exposure duration (with standard deviation), dose rate (dose per 1 min), and mean (with corresponding percentage of the total near-field dose), minimum, median and maximum of the daily cumulative dose for the *brain* and *whole-body* exposure for the near-field predictors.

Near-Field Predictor	Brain SAR (mW/kg)	Whole-Body SAR (mW/kg)	Exposure Duration (min/day)	Brain Dose Rate (mJ/kg/min)	Whole-Body Dose Rate (mJ/kg/min)	Brain Dose (mJ/kg/day)				Whole-Body Dose (mJ/kg/day)			
	Value	Value	Mean (SD)	Value	Value	Mean (%)	Min	Median	Max	Mean (%)	Min	Median	Max
GSM ^1^ mobile phone calls without headset	3.198	0.411	7.6 (13.0)	191.88	24.66	−	−	−	−	−	−	−	−
GSM ^1^ mobile phone calls with headset	0.003198	0.411	1.9 (7.6)	0.19	24.66	−	−	−	−	−	−	−	−
GSM ^1^ mobile phone calls headset considered ^2^	−	−	9.5 (16.7)	−	−	1451.78 (94.6%)	0.00	601.90	22587.02	234.47 (73.3%)	0.00	85.14	3785.98
UMTS mobile phone calls without headset	0.023	0.003	5.8 (14.8)	1.38	0.18	−	−	−	−	−	−	−	−
UMTS mobile phone calls with headset	0.000023	0.003	1.9 (8.1)	0.001	0.18	−	−	−	−	−	−	−	−
UMTS mobile phone calls headset considered ^2^	−	−	7.7 (19.9)	−	−	8.04 (0.5%)	0.00	2.57	217.49	1.39 (0.4%)	0.00	0.37	34.20
DECT phone calls without eco mode	0.373	0.051	−	22.38	3.06	−	−	−	−	−	−	−	−
DECT phone calls with eco mode	0.0373	0.0051	−	2.24	0.31	−	−	−	−	−	−	−	−
DECT phone calls eco mode considered ^3^	−	−	9.0 (10.9)	−	−	74.10 (4.8%)	0.00	18.70	1364.86	10.13 (3.2%)	0.00	2.61	190.28
Mobile phone data traffic	0.000092	0.012	11.5 (22.5)	0.01	0.72	0.06 (0.004%)	0.00	0.01	0.54	8.29 (2.6%)	0.00	1.63	70.89
Mobile phone close to the body (passive data traffic) ^4^	0.000092	0.012	265.2 (349.5)	0.00006	0.01	0.01 (0.001%)	0.00	0.01	0.08	1.91 (0.6%)	0.00	0.86	10.37
Mobile phone data traffic WLAN	0.000092	0.012	30.6 (35.0)	0.01	0.72	0.17 (0.01%)	0.00	0.10	0.54	22.03 (6.9%)	0.00	13.68	70.89
Computer, laptop and tablet use with WLAN	0.000092	0.012	57.6 (83.3)	0.01	0.72	0.32 (0.02%)	0.00	0.17	3.42	41.46 (13.0%)	0.00	21.60	446.40

**^1^** For calls with the mobile phone on the GSM network the mean of the SARs for the GSM900 and the GSM1800 network was used because there was no differentiation between GSM900 and GSM1800 network in the mobile phone operator data; **^2^** Headset considered means that the proportion of headset use was applied to the mobile phone call duration; **^3^** Eco mode of the DECT phone at home was considered for all calls if the DECT phone at home was equipped with eco mode and for no calls if the DECT phone at home had no eco mode; **^4^** A transmission of data for 0.01*exposure duration of carrying the mobile phone close to the body was assumed.

### 3.2. Far-Field Dose

#### 3.2.1. Far-Field Predictors

Mean modelled downlink exposure of the HERMES study participants was 15.8 μW/m² (range: 0.0–476.9 μW/m²) at home and 10.4 μW/m² (0.003–67.1 μW/m²) in school (for details see Supplementary Table S1–Table S4). Mean values for radio broadcasting were 1.7 μW/m² (0.0–40.8 μW/m²) at home and 0.8 μW/m² (0.0–5.1 μW/m²) in school. For TV broadcasting modelled exposure was on average 0.5 μW/m² (0.0–32.1 μW/m²) at home and 0.06 μW/m² (0.0–0.7 μW/m²) in school. In other places (outdoors, in trains, buses and cars, and on locations not further defined in the diary) exposure, obtained from the personal measurements, was on average 46.2 μW/m² for downlink and 5.9 μW/m² for TV. Radio exposure was not measured by the used exposimeters and therefore taken into account only at home and in school. Identification of far-field predictors by multivariable regression models was based on personal measurements for 23.0–121.2 h (measurement duration of 71.2 h on average) from 95 HERMES participants. 

When comparing personal measurements with modelling building characteristics, wall and window frame material, window glazing, window and building age, and façade renovation were not found to modify personal radio, TV or downlink indoor exposure at home or in school. Therefore, we did not take into account any building characteristics.

For the DECT far-field exposure no explanatory variable was associated with the measured DECT exposure from the personal measurements. For that reason, we decided to use the DECT measurements without modification using the average DECT exposure at home from the personal measurements which was 1.18 μW/m².

The identified far-field predictors for the WLAN and the uplink far-field exposure together with the derived exposure contribution per day were:

- Availability of WLAN in school: +0.49 μW/m² (WLAN);
- Availability of WLAN at home and not switching off the base station during night: +1.02 μW/m² (WLAN);
- Number of smartphones used at home: +9.39 μW/m² per smartphone (Uplink);
- Time spent in trains: +0.07 μW/m² per minute spent in trains (WLAN), +1.06 μW/m² per minute spent in trains (Uplink);
- Time spent in buses: +0.64 μW/m² per minute spent in buses (Uplink).

For details see Supplementary Material 1.

#### 3.2.2. Far-Field Dose

**Figure 2:**
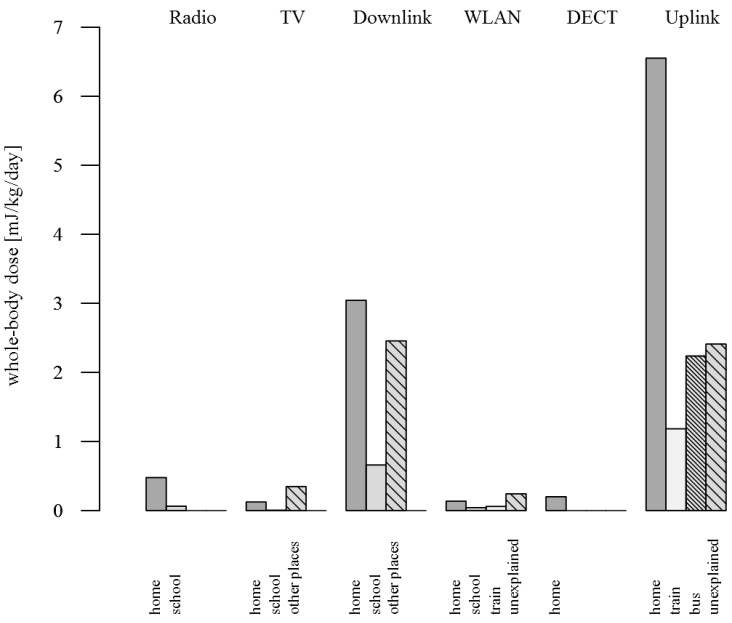
Mean daily cumulative *whole-body* dose for the far-field exposure at different locations; The same pattern was found for the *brain* dose.

The cumulative dose was highest for downlink and uplink for both brain and whole-body dose, whereas dose contributions from radio, TV, WLAN and DECT were small compared to the contributions from downlink and uplink (Table 4 and Table 5). The downlink dose was 8.43 mJ/kg per day (33.5%) for the brain and 6.16 mJ/kg per day (30.4%) for the whole-body. The uplink dose was 15.22 mJ/kg per day (60.4%) for the brain and 12.38 mJ/kg per day (61.2%) for the whole-body. It was mainly the exposure at home and other places (outdoors, in trains, buses and cars and locations not further defined in the diaries) that contributed to the downlink exposure (Figure 2). Being at home and, to a smaller extent, spending time in trains and buses contributed to the uplink exposure whereas a considerable part remained unexplained.

**Table 4:** *Brain* SAR, mean and derivation of the power flux density, *brain* dose rate (dose per 1 min) and mean (with the corresponding percentage of the total brain far-field dose), minimum, median and maximum of the *brain* dose for the far-field exposure.

Band	Description	SAR ((mW/kg)/(mW/m²))	Power Flux Density (mW/m²)		Dose Rate ((mJ/kg)/(mW/m²)/min)	Dose (mJ/kg/day)			
			Mean	Derivation		Mean (%)	Min	Median	Max
Radio ^1^	Radio broadcast transmitter	0.001	0.002	modelling	0.09	0.16 (0.6%)	0.00	0.07	3.30
TV	Television broadcast transmitter	0.008	0.001	modelling and personal measurements	0.46	0.79 (3.1%)	0.58	0.58	14.40
Downlink 900	Transmission from base station to mobile phone handset	0.007	−	−	0.41	−	−	−	−
Downlink 1800	Transmission from base station to mobile phone handset	0.003	−	−	0.19	−	−	−	−
Downlink 2100	Transmission from base station to mobile phone handset	0.003	−	−	0.17	−	−	−	−
Downlink	Downlink 900+ Downlink 1800+ Downlink 2100	−	0.019	modelling and personal measurements	−	8.43 (33.5%)	3.76	5.02	124.64
WLAN	Wireless local area network	0.002	0.002	prediction regression model	0.14	0.39 (1.6%)	0.20	0.40	2.37
DECT	Digital enhanced cordless telecommunications	0.003	0.001	personal measurements	0.17	0.19 (0.8%)	0.19	0.19	0.19
Uplink ^2^	Transmission from mobile phone handset to base station	0.004	0.041	prediction regression model	0.26	15.22 (60.4%)	2.96	13.54	71.16

**^1^** Radio = radio FM (Frequency Modulation) + DAB (Digital Audio Broadcasting); Radio was considered only at home and in school (geospatial propagation modelling) because used exposimeters did not measure radio broadcasting; **^2^** Uplink = Uplink 900+ Uplink 1800+ Uplink 1900; For the far-field uplink exposure from other mobile phones the average of the SARs for the downlink bands downlink 900, downlink 1800 and downlink 2100 was used.

**Table 5:** *Whole-body* SAR, mean and derivation of the power flux density, *whole-body* dose rate (dose per 1min) and mean (with the corresponding percentage of the total whole-body far-field dose), minimum, median and maximum of the *whole-body* dose for the far-field exposure.

Band	Description	SAR((mW/kg)/(mW/m²))	Power Flux Density(mW/m²)		Dose Rate((mJ/kg)/(mW/m²)/min)	Dose(mJ/kg/day)			
			Mean	Derivation		Mean (%)	Min	Median	Max
Radio ^1^	Radio broadcast transmitter	0.005	0.002	modelling	0.29	0.54 (2.7%)	0.00	0.22	11.30
TV	Television broadcast transmitter	0.005	0.001	modelling and personal measurements	0.27	0.47 (2.3%)	0.35	0.35	8.61
Downlink 900	Transmission from base station to mobile phone handset	0.004	−	−	0.26	−-	−	−	−
Downlink 1800	Transmission from base station to mobile phone handset	0.003	−	−	0.20	−	−	−	−
Downlink 2100	Transmission from base station to mobile phone handset	0.003	−	−	0.18	−	−	−	−
Downlink	Downlink 900+ Downlink 1800+ Downlink 2100	−	0.019	modelling and personal measurements	−	6.16 (30.4%)	2.46	3.47	86.19
WLAN	Wireless local area network	0.003	0.002	prediction regression model	0.17	0.48 (2.4%)	0.24	0.49	2.90
DECT	Digital enhanced cordless telecommunications	0.003	0.001	personal measurements	0.18	0.20 (1.0%)	0.20	0.20	0.20
Uplink ^2^	Transmission from mobile phone handset to base station	0.004	0.041	prediction regression model	0.21	12.38 (61.2%)	2.41	11.01	57.87

**^1^** Radio = radio FM (Frequency Modulation) + DAB (Digital Audio Broadcasting); Radio was considered only at home and in school (geospatial propagation modelling) because used exposimeters did not measure radio broadcasting; **^2^** Uplink = Uplink 900+ Uplink 1800+ Uplink 1900; For the far-field uplink exposure from other mobile phones the average of the SARs for the downlink bands downlink 900, downlink 1800 and downlink 2100 was used.

### 3.3. Combining Near-Field and Far-Field Dose

**Figure 3:**
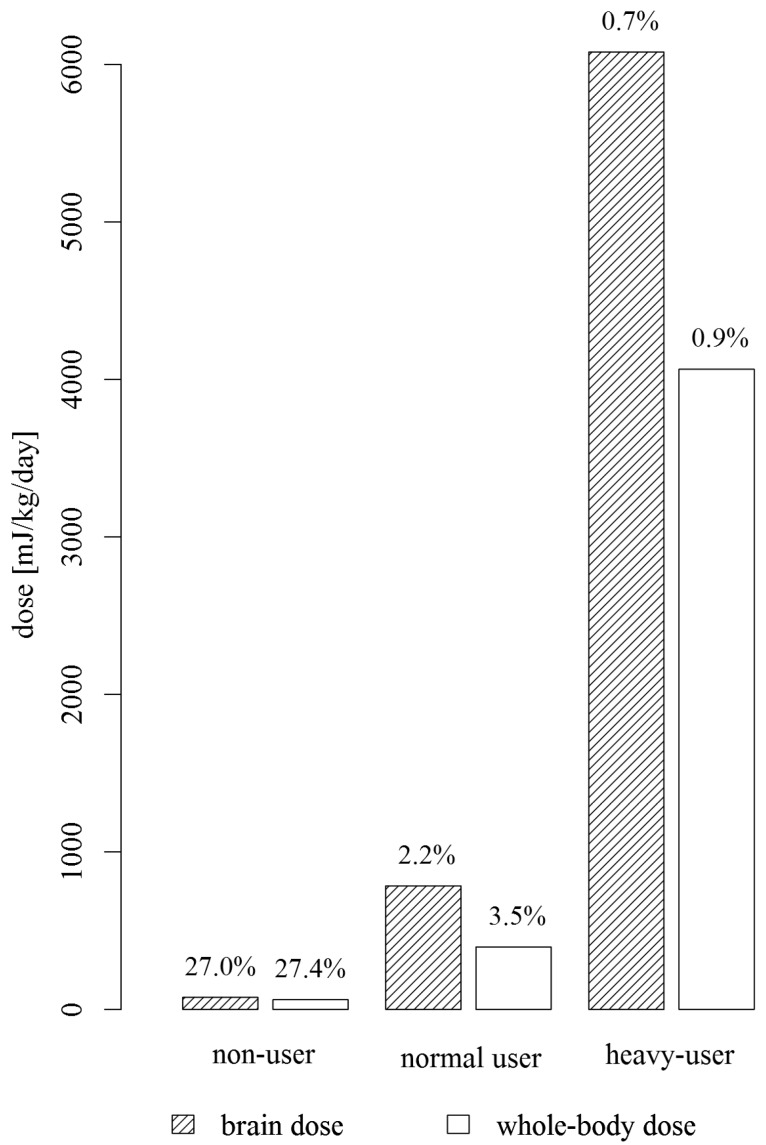
Total brain and whole-body dose for the three HERMES study participants (non-user, normal user, heavy-user); Percentages of the far-field dose on the total dose are indicated above the bars.

The mean brain dose for the HERMES study participants was 1559.7 mJ/kg per day (range: 13.3–22,607.6 mJ/kg/day) whereas the mean whole-body dose was 339.9 mJ/kg per day (6.5–4064.7 mJ/kg/day). The near-field component counted on average for far the most of the total dose, 98.4% (1534.5 mJ/kg/day) of the total brain dose and 94.0% (319.7 mJ/kg/day) of the total whole-body dose originated from near-field sources. For the three HERMES study participants, a non-user, a normal user and a heavy-user, considerable differences in the cumulative dose and in the proportion of the far-field dose on the total dose were found (Figure 3).

Total brain white matter dose was on average 535.0 mJ/kg per day. This corresponded to 34.3% of the total average brain gray matter dose (1559.7 mJ/kg/day). The proportional contributions of the particular near-field exposure predictors and far-field bands were similar to the brain gray matter dose. The proportion of the near-field dose on the total dose was similar as well (98.4%).

### 3.4. Comparing Dose Calculations and Personal Measurements

**Figure 4:**
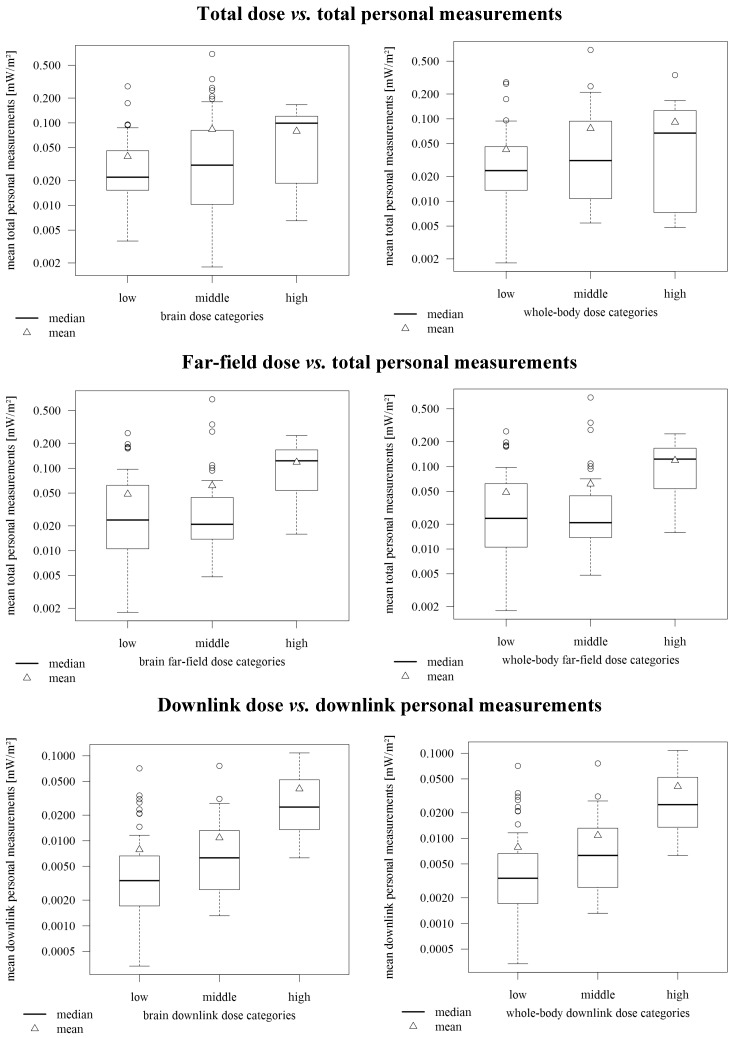
Comparison of predicted dose measures and personal measurements using the three dose categories <50th percentile (low), 50th–90th percentile (medium) and >90th percentile (high); First row: total dose *vs.* total personal measurements; Second row: far-field dose *vs.* total personal measurements; Third row: downlink dose *vs.* downlink personal measurements.

In Figure 4 dose predictions are compared with personal measurements. With respect to total dose (first row of Figure 4) there was a slight tendency that the group median of the personal measurements increased with increasing predicted dose. The Spearman correlation between the dose and the mean of the personal measurements was 0.10 (*p*-value = 0.34) for the brain dose and 0.05 (*p*-value = 0.63) for the whole-body dose. For the far-field dose the picture was similar, but with a slightly higher correlation of 0.18 (*p*-value = 0.08) for the brain far-field dose and 0.17 (*p*-value = 0.09) for the whole-body far-field dose (second row of Figure 4). If taking into account only the downlink dose and the downlink measurements (third row of Figure 4) the mean and the median of the measurements were clearly increased for increasing predicted dose. The Spearman correlation was 0.53 (*p*-value < 0.0001) for the brain downlink dose and 0.52 (*p*-value < 0.0001) for the whole-body downlink dose.

## 4. Discussion

The aim was to develop an integrative exposure surrogate consisting of a near-field and a far-field component representing together total personal RF-EMF dose. Thus we combined near-field exposure from the use of wireless communication devices and far-field exposure from environmental sources such as fixed site transmitters, WLAN and DECT base stations and other people’s mobile phones in the surroundings to one single RF-EMF exposure measure.

### 4.1. Near-Field Exposure

We found GSM mobile phone calls contribute by far the most to the near-field exposure from the use of wireless communication devices. For the brain exposure, DECT phone calls and to a less extent UMTS mobile phone calls contributed as well. Mobile phone data traffic and computer, laptop and tablet use with WLAN played a minor role. For the whole-body exposure computer, laptop and tablet use with WLAN and mobile phone data traffic via WLAN contributed as well, followed by DECT phone calls and mobile phone data traffic via mobile internet connection. UMTS mobile phone calls played a minor role.

### 4.2. Far-Field Exposure

Far-field exposures from radio and TV broadcast transmitters and mobile phone base stations were estimated using geospatial propagation modelling. We did not find any influence of building characteristics on the personal measurements taken at home and in school. This is in contrast to our previous study where metal window frames and concrete walls resulted in a significant exposure reduction [14]. However, our finding is in line with a recent study on modelled mobile phone downlink exposure in the city of Amsterdam, Netherlands, where none of the building characteristics could explain additional variance of the modelled values [26]. We found the availability of WLAN at home and not switching off the base station during night and the availability of WLAN in school being relevant exposure predictors. Furthermore, the time spent in trains explained part of the measured WLAN exposure. Because of the increase of WLAN in public spaces and public transport this part of WLAN exposure may become even more important in the future. The number of smartphones being used at home was the strongest predictor for the far-field uplink exposure followed by the time spent in trains and buses. A considerable part of the uplink exposure however remained unexplained. Previous studies have also demonstrated high uplink exposure in public transport [27,28,29] or investigated the influence of small cells in trains on the exposure of mobile phone users [30]. The relevance of mobile phones in stand-by mode for exposure is still quite unclear. Urbinello *et al.* demonstrated that personal RF-EMF exposure was affected by one’s own mobile phone in stand-by mode because of its regular location updates and push functions implemented in applications [29]. This finding may explain why the number of smartphones at home is one of the exposure relevant predictors. And, additionally, this finding led us to include passive mobile phone data traffic for the near-field exposure estimate. Carrying a mobile phone on the body contributed on average 0.56% to the total whole-body exposure of the HERMES participants.

The contribution of the far-field exposure is small compared to the contribution of the near-field exposure (1.6% of the brain dose and 6.0% of the whole-body dose originated from far-field sources). Nevertheless, far-field exposure is relevant: There are public concerns about potential health effects related to mobile phone base stations [31] and exposure from broadcast transmitters and mobile phone base stations is not lifestyle related which complicates the investigation of soft outcomes (e.g., symptoms and behaviour). Furthermore, far-field exposure is long-term and continuous and people are exposed during night as well, which might be a critical time window. Therefore we think it is worth the effort to investigate far-field exposure as well.

### 4.3. Comparing Dose Calculations and Personal Measurements

In our exposure assessment approach we combined questionnaire data, operator data, modelling and personal measurements from a subsample. This is more efficient than conducting personal measurements in a large sample which is very time- and resource-consuming. Furthermore, near-field exposure from the use of wireless communication devices is not recorded adequately by personal measurements because the measured values depend highly on the distance between the emitting device and the exposimeter, which is not necessarily the same as the distance between the emitting device and the body [4,5]. Only the latter is relevant for exposure. This may also explain why we found only a small correlation between predicted brain and whole-body exposure and personal measurements (Figure 4). For both exposure proxies, the brain dose and the whole-body dose, GSM mobile phone calls are most relevant, but the resulting exposure is not measured accurately with exposimeters during personal measurements [4,5]. However, the predicted far-field dose was also only weakly correlated with personal measurements. This may have several reasons. First, radio broadcasting is not measured by the exposimeters used but modelled at home and in school and thus considered for the dose. Nevertheless, this contribution is small and cannot explain the discrepancy. Second, the prediction models for the WLAN and uplink far-field exposure have limited explanatory power and for DECT no exposure predictor could be identified at all. Thus, there is more work needed to figure out what predictors are able to predict these exposures in a more accurate way. Strikingly, the downlink dose and the personal downlink exposure measurements correlated well. Thus, modelled exposure at home and in school may well be used to predict downlink exposure.

Obviously, the dose calculations are subject to a large uncertainty. We relied our calculations on self-reported amount of mobile phone use, which is typically overestimated by adolescents [32,33]. In our study, overestimation was on average by a factor of 9.3 according to a comparison with operator recorded duration of mobile phone calls. Subsequent dose estimations for our study sample with operator recorded mobile phone data yielded on average a brain gray matter dose of 139.3 mJ/kg per day and a whole-body dose of 24.9 mJ/kg per day for mobile phone calls (brain dose of 1459.8 mJ/kg/day and whole-body dose of 235.9 mJ/kg/day for self-reported duration of mobile phone calls). For the normal user, the proportion of the far-field dose on the total dose was 9.4% for the brain dose and 4.3% for the whole-body dose if taking into account operator recorded duration of mobile phone calls (2.2% and 3.5% for the brain and the whole-body dose, respectively for self-reported duration of mobile phone calls, Figure 3).

We have obtained SAR values from the literature, however, such data are still rare and have a large uncertainty range. Unfortunately, systematic analyses of this uncertainty are not yet published and could thus not be considered in our study. Most of the uncertainty is due to the unknown position of the device in relation to the body. Ideally, this should be measured permanently for each study participant. However, this is impossible and one has thus to rely on assumptions about typical positions. A further source of uncertainty is the emitted output power of mobile phones, in particular during data transmission and in stand-by mode. Depending on the type of data transmission (e.g., watching videos and playing games while connected to the internet, using social networks and reading news), a mobile phone may mainly act as receiver or transmitter. We did not find any data about proportion of time the mobile phone is transmitting data when set in stand-by mode, and which factors are relevant for these emissions. Additional uncertainty remains regarding SARs for newer devices such as tablets. Due to lack of data, we did not take into account exposure from use of the mobile phone as mobile hotspot. Inherent uncertainties are related to the geospatial propagation modelling and predictions derived from the personal measurements. Also the assessment of the proportion of calls made on the GSM and UMTS network comes with uncertainties. 

## 5. Conclusions

Despite all these uncertainties and limitations, the proposed approach is considered useful to combine near-field and far-field exposure to one single integrative exposure surrogate either for the whole-body or for specific organs. However, more work is needed to deepen the understanding of far-field exposure predictors on one hand and near-field exposure from rapidly developing devices such as smartphones and tablets on the other hand. If this approach is refined, the integrative exposure surrogate can be adapted to any population of epidemiologic studies if modelled RF-EMF exposure from fixed site transmitters for the study area, operator data including type of network and specific questionnaire data are available.

## Supplementary Files

Supplementary File 1
