# Supplementary material for: Development of an RF-EMF Exposure Surrogate for Epidemiologic Research
Source: Int J Environ Res Public Health. 2015 May 22;12(5):5634–56. doi: 10.3390/ijerph120505634 (PMC4454989; doi:10.3390/ijerph120505634)
Supplement: Supplementary File 1 [file ijerph-12-05634-s001.pdf]

# Development of an RF-EMF Exposure Surrogate for Epidemiologic Research

**Table S1.** WLAN prediction model: predicted far-field WLAN exposure contribution from a multivariable regression model using bootstrap (1000 replications), n = 95.

| Mean WLAN Personal Measurements (µW/m²) | Exposure Contribution (µW/m²) | 95% Confidence Interval | *p*-Value |
|---|---|---|---|
| WLAN in school | 0.49 | (−1.29, 2.26) | 0.589 |
| WLAN at home and not switched off during night | 1.02 | (−0.80, 2.83) | 0.272 |
| Time spent in trains (min/day) | 0.07 | (−0.06, 0.19) | 0.290 |
| Unexplained by the model | 1.00 | (−0.51, 2.52) | 0.195 |

**Table S2.** Uplink prediction model: predicted far-field uplink exposure contribution from a multivariable regression model using bootstrap (1000 replications), n = 95.

| Mean Uplink Personal Measurements (µW/m²) | Exposure Contribution (µW/m²) | 95% Confidence Interval | *p*-Value |
|---|---|---|---|
| Number of smartphones at home | 9.39 | (−6.33, 25.12) | 0.242 |
| Time spent in trains (min/day) | 1.06 | (0.24, 1.87) | 0.011 |
| Time spent in buses (min/day) | 0.64 | (−0.02, 1.30) | 0.057 |
| Unexplained by the model | 7.89 | (−18.40, 34.18) | 0.556 |

**Table S3.** Mean exposure duration (per day) of the different locations for the calculation of the far-field dose.

| Location | Mean Exposure Duration (per day) |
|---|---|
| Home day [1] | 8 h 21 min |
| Home night [1] | 7 h 21 min |
| Home | 15 h 41 min |
| School | 4 h 43 min |
| Outside | 1 h 41 min |
| Train | 0 h 6 min |
| Bus | 0 h 9 min |
| Car | 0 h 13 min |
| Unspecified location [2] | 1 h 27 min |

[1] Home day and night: Home day means the time being at home in the time period 6:00 until 22:00; Home night means the time being at home in the time period 22:00 until 6:00; [2] Unspecified location means diary entries recorded as miscellaneous or other activity or location than the prespecified activities and locations in the time-activity diary.

**Table S4.** Measured, modelled or predicted mean power flux densities ($\mu W/m^2$) at the different locations for the calculation of the far-field dose.

| Band | Description | Power Flux Density ($\mu W/m^2$) | | | | | | | | | |
|---|---|---|---|---|---|---|---|---|---|---|---|
| | | Home Day [2] | Home Night [2] | Home | School | Outside | Train | Bus | Car | Unspecified Location [3] | Unexplained [4] |
| Radio [1] | Radio broadcast transmitter | 1.73 | 1.73 | – | 0.77 | – | – | – | – | – | – |
| TV | Television broadcast transmitter | 0.48 | 0.48 | – | 0.06 | 4.14 | 7.25 | 24.70 | 4.38 | 6.16 | – |
| Downlink 900 | Transmission from base station to mobile phone handset | 6.52 | 4.32 | – | 4.98 | 35.80 | 64.30 | 31.60 | 16.60 | 27.60 | – |
| Downlink 1800 | Transmission from base station to mobile phone handset | 5.15 | 3.97 | – | 3.56 | 5.18 | 8.49 | 7.80 | 5.09 | 9.89 | – |
| Downlink 2100 | Transmission from base station to mobile phone handset | 5.81 | 4.15 | – | 1.90 | 6.88 | 15.20 | 16.30 | 3.21 | 6.09 | – |
| WLAN | Wireless local area network | – | – | 0.56 | 0.18 | – | 0.24 | – | – | – | 1.00 |
| DECT | Digital enhanced cordless telecommunications | – | – | 1.18 | – | – | - | – | – | – | – |
| Uplink [5] | Transmission from mobile phone handset to base station | – | – | 21.48 | – | – | 3.88 | 7.33 | – | – | 7.89 |

For RF-EMF a power flux density of 1 $\mu W/m^2$ = 0.001 $mW/m^2$ corresponds to an electric field strength of 0.019 V/m; [1] Radio = radio FM (Frequency Modulation) + DAB (Digital Audio Broadcasting); Radio was considered only at home and in school (geospatial propagation modelling) because used exposimeters did not measure radio broadcasting; [2] Home day and night: Home day means the time being at home in the time period 6:00 until 22:00; Home night means the time being at home in the time period 22:00 until 6:00; [3] Unspecified location means diary entries recorded as miscellaneous or other activity or location than the prespecified activities and locations in the time-activity diary; [4] Unexplained means the part of the predicted WLAN and uplink power flux density not explained through the relevant far-field predictors found in the multivariable regression model used to predict WLAN and uplink exposure, respectively; [5] Uplink = Uplink 900 + Uplink 1800 + Uplink 1900.

© 2015 by the authors; licensee MDPI, Basel, Switzerland. This article is an open access article distributed under the terms and conditions of the Creative Commons Attribution license (http://creativecommons.org/licenses/by/4.0/).
